# Proteomics of Aqueous Humor as a Source of Disease Biomarkers in Retinoblastoma

**DOI:** 10.3390/ijms232113458

**Published:** 2022-11-03

**Authors:** Angela Galardi, Christina Stathopoulos, Marta Colletti, Chiara Lavarello, Ida Russo, Raffaele Cozza, Antonino Romanzo, Angel M. Carcaboso, Franco Locatelli, Andrea Petretto, Francis L. Munier, Angela Di Giannatale

**Affiliations:** 1 Department of Pediatric Hematology/Oncology and Cell and Gene Therapy, IRCCS, Ospedale Pediatrico Bambino Gesù, Piazza di Sant’ Onofrio 4, 00165 Rome, Italy; 2 Jules Gonin Eye Hospital, Fondation Asile des Aveugles, University of Lausanne, 1002 Lausanne, Switzerland; 3 Core Facilities-Clinical Proteomics and Metabolomics, IRCCS, Istituto Giannina Gaslini, Via Gerolamo Gaslini 5, 16147 Genoa, Italy; 4 Ophtalmology Unit, IRCCS, Ospedale Pediatrico Bambino Gesù, Piazza Sant’Onofrio 4, 00165 Rome, Italy; 5 SJD Pediatric Cancer Center Barcelona, Hospital Sant Joan de Deu, Institut de Recerca Sant Joan de Deu, Esplugues de Llobregat, 08950 Barcelona, Spain; 6 Department of Life Sciences and Public Health, Catholic University of the Sacred Heart, 00168 Rome, Italy

**Keywords:** retinoblastoma, aqueous humor, proteomic, tumor biomarker

## Abstract

Aqueous humor (AH) can be easily and safely used to evaluate disease-specific biomarkers in ocular disease. The aim of this study was to identify specific proteins biomarkers in the AH of retinoblastoma (RB) patients at various stages of the disease. We analyzed the proteome of 53 AH samples using high-resolution mass spectrometry. We grouped the samples according to active vitreous seeding (Group 1), active aqueous seeding (Group 2), naive RB (group 3), inactive RB (group 4), and congenital cataracts as the control (Group 5). We found a total of 889 proteins in all samples. Comparative parametric analyses among the different groups revealed three additional proteins expressed in the RB groups that were not expressed in the control group. These were histone H2B type 2-E (HISTH2B2E), InaD-like protein (PATJ), and ubiquitin conjugating enzyme E2 V1 (UBE2V1). Upon processing the data of our study with the OpenTarget Tool software, we found that glyceraldehyde 3-phosphate dehydrogenase (GAPDH) and CD44 were more highly expressed in the RB groups. Our results provide a proteome database regarding AH related to RB disease that may be used as a source of biomarkers. Further prospective studies should validate our finding in a large cohort of RB patients.

## 1. Introduction

Retinoblastoma (RB) is the most frequent eye cancer worldwide, representing 6% of all pediatric cancers in children under age 5 [1]. The tumor is caused by the inactivation of the gene *RB1*, a tumor suppressor gene located on chromosome 13q14 [2], and it arises from immature retinal cells. While growing, it has the capacity to seed and survive in other non-vascularized eye compartments, such the vitreous body and the aqueous humor (AH), and/or to metastasize outside the eye. The mechanisms of RB tumorigenesis and its progression are still not fully elucidated.

Until recently, molecular characterization of RB was exclusively performed on tumor samples derived from enucleation, as traditional biopsy remained contraindicated by fear of tumor spread. Recent advances in the treatment of patients affected by RB have allowed for the safe extraction of AH by paracentesis [3]. This procedure paved the way obtaining a timely correlation between AH analysis and disease evolution. In recent years, several studies have reported that cell free DNA isolated from the AH of enucleated or conservatively treated eyes with RB can be used to characterize the genetics of the disease [4,5,6,7,8,9,10,11,12,13], and it may also correlate with clinical outcomes [14,15,16]. On the other hand, the modulation of several proteins has been described in AH obtained after enucleation [17,18,19,20,21,22] or collected from patients who underwent intravitreal chemotherapy [23].

In the present study, we present a proteomic analysis of 53 AH samples obtained from eyes with RB at various stages of the disease, as well as eyes with congenital cataracts, with the aim of identifying specific protein biomarkers of the disease.

## 2. Results

### 2.1. Proteomic Characterization of AH Samples

To identify potential biomarkers related to the disease status in patients affected by RB, we have analyzed 53 AH samples derived from 45 eyes and 44 different patients. These samples were subdivided into 5 subgroups according to the function of the disease characteristics at the time of the AH collection These included 5 samples from 5 children with isolated vitreous seeding relapse (Group 1), 9 samples from 2 children with active aqueous seeding confirmed on cytopathology (Group 2), 6 samples from 6 children with naïve retinoblastoma, but no aqueous seeding involvement (Group 3), 27 samples from 26 children with inactive retinoblastoma (Group 4), and 6 samples from 5 children with congenital cataracts used as controls (Group 5).

**Figure 1:**
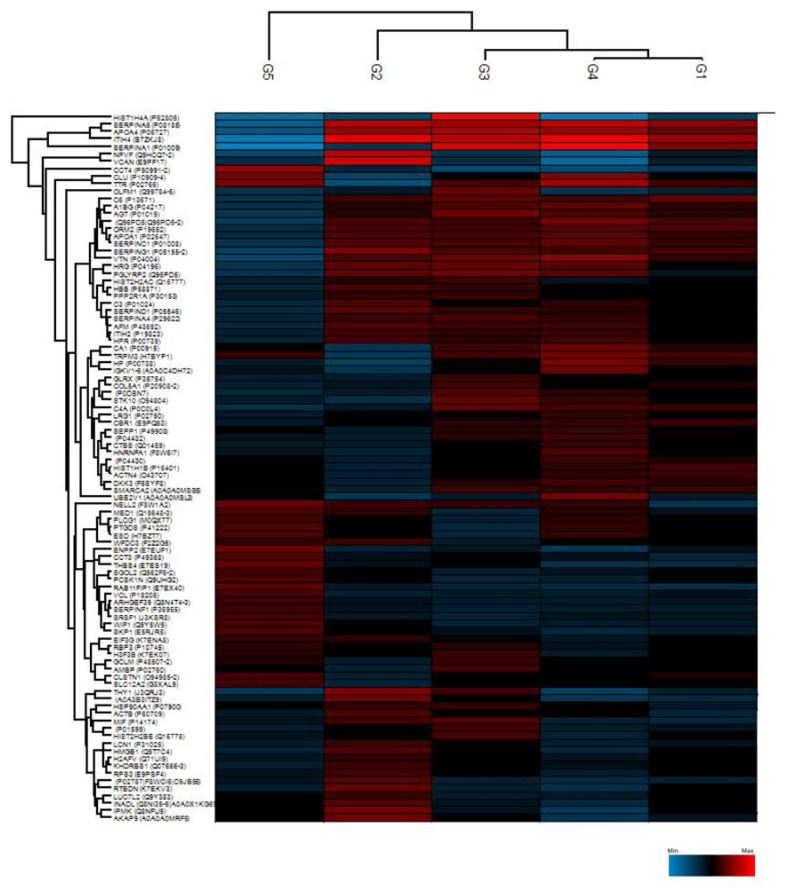
Unsupervised hierarchical-clustered heatmap of proteins identified by ANOVA testing. Heatmap showing the distinctive proteomic signature of the 5 different groups: Group 1 (active vitreous seeding), Group 2 (active aqueous seeding), Group 3 (naïve RB), Group 4 (inactive RB), and Group 5 (congenital cataracts). The amount of each protein in the different groups is represented by the color scheme, in which red and blue indicate high and low proteins expression, respectively.

The proteomic analysis of all 53 AH samples identified a total of 889 proteins. The proteins were then filtered to have a 70% presence in at least one of the groups. The expression matrix was divided into groups of proteins according to their abundance. Unsupervised clustering of the 53 individual AH samples revealed distinctive clustering within the different groups (Figure 1). The heatmap built based on the biological processes shows that several of the upregulated proteins in Group 1 (active vitreous seeding) are involved in transport, immune response and protein metabolism. Upregulated proteins in Group 2 (active aqueous seeding) are involved in protein metabolism, while upregulated proteins in Group 3 (naïve RB) are involved in the regulation of nucleoside synthesis. Finally, most of the upregulated proteins in Group 4 (inactive RB) are involved in the energy pathway and protein metabolism. Group 5 (congenital cataracts) also shows abundant proteins involved in protein metabolism, among which three proteins—corticosteroid-binding globulin-SERPINA6, apolipoprotein A-IV (APOA4), and inter-alpha-trypsin inhibitor heavy chain H4 (ITIH4)—were downregulated compared to other groups.

### 2.2. Student’s t-Test Analysis

**Figure 2:**
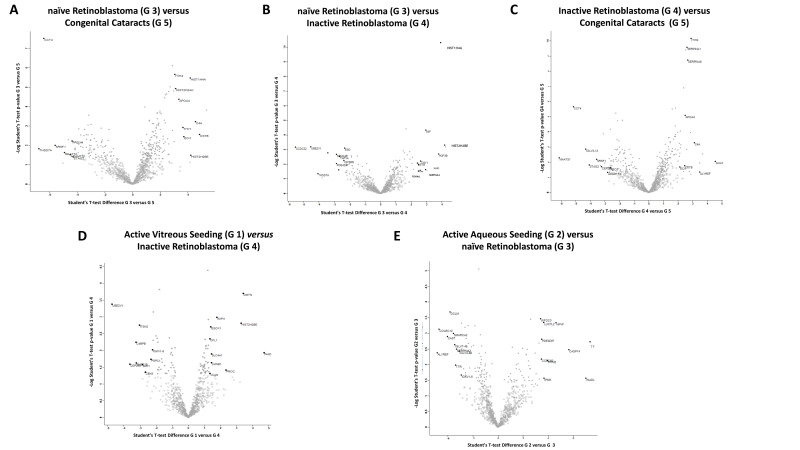
Volcano plots showing abundance of differentially expressed proteins between two of the five groups at a time: (**A**) naïve RB compared to congenital cataracts; (**B**) naïve *RB* compared to inactive RB; (**C**) inactive RB compared to congenital cataracts; (**D**) active vitreous seeding compared to inactive RB; (**E**) active aqueous seeding compared to naïve RB. The black line shows *p* value = 0.05 and s0 = 0.1. Red dots depict proteins whose fold change is <2 (log2 = 1) or *p* > 0.05 between the two groups considered.

Student’s t-test analysis (*p* value 0.05 and S0 > 0.1,) was used to identify the 10 statistically significantly most upregulated proteins between the different groups by comparing two groups at a time. The significant proteins were plotted using a volcano plot for each comparison (Figure 2).

#### 2.2.1. Differentially Expressed Proteins in Group 3 (Naïve RB) Compared to Group 5 (Congenital Cataracts)

From the comparison of the protein expression between Group 3 and Group 5, the 10 significantly most upregulated proteins in Group 3 were: tropomyosin alpha-4 chain (TPM4); cystatin-B (CSTB); complement C4-A (C4A); histone H2B type 2-E (HIST2H2BE); histone H4 (HIST1H4A); THO complex subunit 4 (ALYREF); enoyl-CoA delta isomerase 1 (ECI1); Thy-1 antigen (THY1); PTB Domain Containing engulfment Adapter protein1 (GULP1); and splicing factor 3A subunit 3 (SF3A3). Three proteins were involved in cytoskeleton remodeling (TPM4; CSTB; GULP1), two in cell metabolism (ALYREF; ECI1) and one in cell adhesion (THY1).

The top 10 significantly most upregulated proteins in Group 5 were: thrombospondin type-1 domain-containing protein 7A (THSD7A); T-complex protein 1 subunit delta (CCT4); serine/arginine-rich-splicing factor 1 (SRSF1); centrosomal protein 295 kDa (CEP295); cilia- and flagella-associated protein 91 (MAATS1); immunoglobulin lambda variable 3–12 (IGLV3-12); TATA element modulatory factor (TMF1); and disintegrin and metalloproteinase with thrombospondin motifs 1 (ADAM-TS 1); peroxiredoxin-4 (PRDX4); ATPase inhibitor (ATP5IF1). Among these proteins, two were involved in protein misfolding and aggregation (TMF1; ATP5IF1), three in ciliogenesis formation (SRSF1; CEP295; MAATS1), and two in extracellular matrix and cytoskeleton organization (THSD7A; ADAMT-S1), (Figure 2A, Supplementary Table S1).

#### 2.2.2. Differentially Expressed Proteins in Group 3 (Naïve RB) Compared to Group 4 (Inactive RB)

From the comparison of the protein expression between Group 3 and Group 4, the 10 significantly most upregulated proteins in Group 3 were: HIST2H2BE; HIST1H4A; histone H3 (H3F3B); macrophage migration inhibitory factor (MIF); immunoglobulin heavy constant delta (IGHD); THY1; Alpha-1-antichymotrypsin (SERPINA3); immunoglobulin lambda variable 3–19 (IGLV3-19); proteasome subunit alpha type (PSMA6); and CSTB. Three proteins (MIF; SERPINA3; PSMA6) play a pro-inflammatory role. Interestingly, four of these ten proteins were also up regulated in the comparison between the Group 3 and the Group 5 (HIST2H2BE, HIST1H4A, THY1, CSTB).

The 10 significantly most upregulated proteins in Group 4 were: coiled-coil domain–containing protein 22 (CCDC22); ubiquitin-conjugating enzyme E2 variant 1 (UBE2V1); THSD7A; caspasi-14 (CASP14); TMF1; semaphorin-4B (SEMA4B); myomegalin (PDE4DIP); immunoglobulin heavy variable 4–34 (IGHV4-34); TBP-associated factor 1A (TAF1A); and IGF-binding protein 6 (IGFBP6). In summary, in treated inactive RB, the proteins were mainly involved in protein catabolism (CCDC22; UBE2V1; TMF1), cytoskeleton remodeling (THSD7A; PDE4DIP), differentiation (CASP14; SEMA4B), transcriptional regulation (TAF1A), and in cell metabolism (IGFBP6), (Figure 2B, Supplementary Table S1).

#### 2.2.3. Differentially Expressed Proteins in Group 4 (Inactive RB) Compared to Group 5 (Congenital Cataracts)

From the comparison of the protein expression between Group 4 and Group 5, the 10 significantly most upregulated proteins in Group 4 were: GULP1; transducin beta chain 3 (GNB3); ALYREF; C4A; ITI heavy chain H4 (ITIH4); nucleolar protein 10 (NOL10); haptoglobin (HP); zinc finger C4H2 domain-containing protein (ZC4H2); corticosteroid-binding globulin (SERPINA6); and serum amyloid A-1 protein (SAA1). These proteins were mainly involved in cell metabolism (GNB3; ALYREF; SERPINA6) and inflammation (C4A; SAA1).

The 10 significantly most upregulated proteins in Group 5 were: MAATS1; CCT4; IGLV3-12; cohesin subunit SA-2 (STAG2); SRSF1; M-phase inducer phosphatase 1 (CDC25A); CEP295; vinculin (VCL); H3F3B; and ADAM-TS 1. The majority of these proteins were involved in ciliogenesis formation (CCT4; CEP295; MAATS1), (Figure 2C, Supplementary Table S1).

#### 2.2.4. Differentially Expressed Proteins in Group 1 (Active Vitreous Seeding) Compared to Group 4 (Inactive RB)

From the comparison of the protein expression between Group 1 and Group 4, the 10 significantly most upregulated proteins in Group 1 were: InaD-like protein (INDAL/PATJ); demantin (DMTN); HIST2H2BE; switch-associated protein 70 (SWAP70); centromere protein F (CENPF); microtubule-associated protein 1A (MAP1A); vitamin K-dependent protein C (PROC); protein SOGA 1 (SOGA1); protein FAM83F (FAM83F); immunoglobulin lampda constant 7 (IGLC7). Six proteins (INDAL/PATJ; DMTN; SWAP70; CENPF; MAP1A; LMNB2) were involved in actin and cytoskeleton rearrangement.

In Group 4, the 10 most significantly upregulated proteins were: UBE2V1; CEP295; C4b-binding protein beta chain (C4BPB); SH3 domain-containing protein 19 (SH3D19); intersectin-2 (ITSN2); osteopontin (SPP1); ZC4H2; chromobox protein homolog 5 (CBX5); dynenin axonemal heavy chain 5 (DNAH5); and protein ELYS (AHCTF1). These proteins were mainly implicated in ciliogenesis, movement (CEP295; C4BPB; DNAH5), and catabolism (UBE2V1) (Figure 2D, Supplementary Table S1).

#### 2.2.5. Differentially Expressed Proteins in Group 2 (Active Aqueous Seeding) Compared to Group 3 (Naïve RB)

From the comparison of the protein expression between Group 2 and Group 3, the 10 significantly most upregulated proteins in Group 2 were: serotransferrin (TF); INADL/PATJ; CASP14; neuropeptide RFFP-3 (NPVF); stromal interaction molecule 2 (STIM2); inositol polyphosphate multikinase (IMPK); putative RNA-binding protein Luc7-like 2 (LUC7L2); PDE4DIP; coiled-coil domain-containing protein 60 (LUC7L2); and WAP four-disulfide core domain protein 3 (WFDC3). Group 2 was enriched in proteins with a role in cytoskeleton rearrangement (InaD-like protein; PDE4DIP), metabolism (IMPK; NPVF), protease activity (TF; WFDC3), calcium homeostasis (STIM1), and differentiation (CASP14).

The 10 significantly most upregulated proteins in Group 3 were: ALYREF; collectin-12 (COLEC12); calpain inhibitor (CAST); glutamate cysteine ligase (GLCM); probable global transcription activator SNF2L2 (SMARCA2); immunoglobulin lambda variable 7–46 (IGLV7-46); transthyretin (TTR); SERPINA3; immunoglobulin lambda variable 4–69 (IGLV4-69); and SH3 domain-containing protein 19 (SH3D19). These proteins were involved in: cellular metabolism (ALYREF; GLCM), cytoskeleton and chromatin remodelling (CAST; SMARCA2), and in the retinol metabolic process (transthyretin) (Figure 2E, Supplementary Table S1).

### 2.3. Identification of RB Related Genes in Our Experimental Matrix Using the OPENTARGET Tool

**Figure 3:**
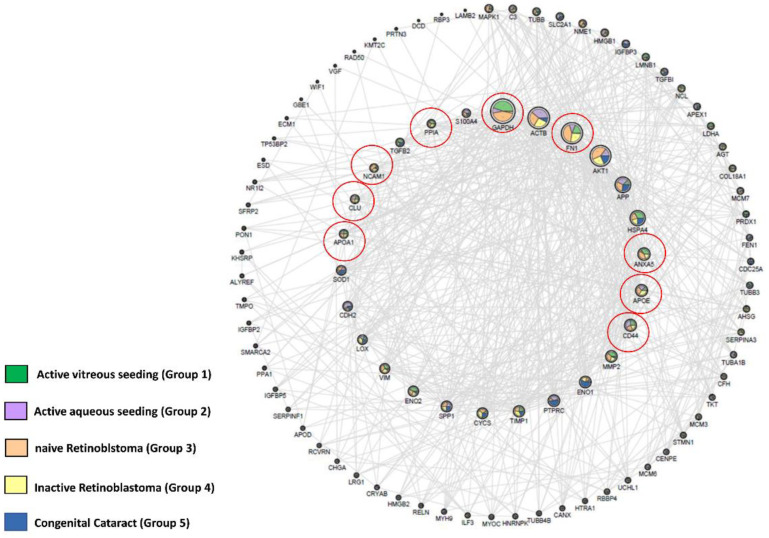
The network represents the interactions between 96 genes coding proteins identified overlapping the polypeptides associated with RB, selected via Open Target, and the proteins identified in our study groups (including the congenital cataract group). The nodes have dimensions related to the degree of connection, and the internal circumference shows the most connected proteins, with a degree > 15. The outer circumference shows the less connected proteins (degree ≤ 15); therefore, of smaller dimensions. Connections are only present if the confidence level of the string is > 0.4. The coloring of the nodes is a function of the average intensity of each group measured in mass spectrometry, normalized for graphic reasons on a scale of 1–100. The coloring and the percentage distribution of the node were preformed using the layout tools in Image/Chart of Cytoscape.

When exploring the proteomic data obtained in our study with Open Target [24], a total of 96 proteins associated with RB could be identified when all groups (including the one with congenital cataracts) were considered. Nine of them were found in RB samples only, namely GAPDH, FN1, ANAXA5, APOE, CD44, APOA1, CLU, NCAM1, and PPIA (Figure 3, Supplementary Table S2).

Glyceraldehyde-3-phosphate dehydrogenase (GAPDH) is an enzyme involved in energy metabolism and in the production of ATP and pyruvate through anaerobic glycolysis in the cytoplasm. GAPDH is one of the major housekeeping proteins. Independent of its role in energy metabolism, GAPDH has other functions, including DNA replication and repair, endocytosis, exocytosis, cytoskeletal organization, iron metabolism, cell death, and carcinogenesis. This protein is upregulated in several cancers, suggesting its involvement in tumor growth. GAPDH interacts with active AKT, sustaining enzyme activity; thus, this protein may be a crucial regulator of cancer cell functions and a marker of cancer cell progression and prognosis [25]. Higher levels of GAPDH are found in diabetic retinopathy; in fact, we can see the nuclear translocation of GAPDH in retinal ganglion cells. This is correlated with damage, along with induced mechanisms of retinal neurodegeneration during diabetic retinopathy development [26].

Annexin A5 (ANXA5) is a phospholipase A2 and protein kinase C inhibitory protein with calcium channel activity, playing an important role in cellular signal transduction, inflammation, growth, and differentiation. Anxa5 is up-regulated in several tumors and is involved in tumor progression, invasion, metastasis, and drug resistance [27]. In the eye, ANXA5 has a crucial function in clearance phagocytosis in the retinal pigment epithelium (RPE) [28].

Fibronectin 1 (FN1) is a member of the glycoprotein family and is widely expressed by multiple cell types. FN1 is involved in cellular adhesion, migration, and differentiation and plays an important role in wound healing and embryonic development. Furthermore, FN1 expression has been associated with cancer progression in several types of tumors. In particular, Bifie Li et al. showed that FN1 expression was upregulated in melanoma metastatic cells, when compared to primary tumor cells [29]. FN1 is a key gene in the RB tumor, and various works have confirmed that FN1 has been recognized to support cell proliferation and migration because it is correlated with MYCN [30]. Interestingly, using SVM analysis, Li et al. identified FN1 among 5 key genes in RB tumors [31].

ApoE and ApoA1 belong to the apolipoproteins family (APOs) and bind to lipids to form lipoproteins. APOA1 and APOE are dysregulated in several tumors and interact with both the innate and adaptive immune system to sustain cancer cell proliferation [32]. These proteins are involved in many intraocular functions by binding to lipids and acting as enzymes in lipid metabolism. In particular, APOA1 and APOE have been shown to be elevated in the AH of primary open angle glaucoma patients as compared to patients with cataracts [33]. The overexpression of APOA1 has been previously documented in RB tumors [34].

CD44 antigen is a cell-surface glycoprotein involved in cell–cell interactions, cell adhesion, and cell migration. Indeed, CD44 binds the hyaluronic acid, inducing cell proliferation and survival, changes on the cytoskeleton, and enhancement of the cellular motility. This protein is thought to play a role in the adaptive plasticity of cancer cells, and its role in EMT has been studied in several cancers, showing a correlation between migration and invasion [35]. Balla et al. showed that the overexpression of CD44 in primary RB tumors may be correlated to the presence of hyaluronic acid in the vitreous fluid [36].

Clusterin (CLU) is a protein implicated in lipid transport, membrane recycling, cell adhesion, programmed cell death, and complement cascade. It is upregulated in many cancers, and its cytoplasmic form (sCLU) is most expressed in aggressive late-stage tumors. Indeed, sCLU is involved in chemoresistance, since it seems to have a key role in preventing apoptosis induced by cytotoxic agents [37]. In particular, CLU, has a protective function in various retinal cells. Song et al. showed that clusterin was highly expressed in human RB tissues and cell lines, demonstrating that its overexpression induced cisplatin resistance [38].

NCAM1 belongs to the immunoglobulin superfamily of adhesion molecules and plays an important role in the regulation of neurogenesis, neurite outgrowth, proliferation, and cell migration [39]. NCAM1 is present in high levels in RB tissue and cell lines, confirming the neuroectodermal origins of this tumor; furthermore, it has been demonstrated to be involved in the immunomodulation of RB [40].

PPIA, or CYPA, is a component of the immunophilin family with peptidyl prolyl cis-trans isomerase (PPIase) activity that regulates protein folding and trafficking. CYPA has been shown to be correlated with different disorders, including cancer, by binding membrane receptors or intracellular partners to activate downstream signaling pathways. It has been described that CYPA can also be secreted by the cells, promoting carcinogenesis, tumor invasion, and drug resistance, and that it may represent a potential circulating biomarker in nasopharyngeal carcinoma [41]. In RB, a direct interaction between CYPA and RB protein has been demonstrated, suggesting its implication in tumor formation [42].

## 3. Discussion

The identification of proteins as biomarkers at different disease stages of RB could allow for a better understanding of the tumorigenesis and assist in disease prognosis, as well as promote the development of targeted therapies. Herein, we present a proteomic study on AH samples obtained from eyes with RB at various stages of the disease. AH samples of congenital cataracts were used as the control group. The differentially upregulated proteins in RB are mainly involved in lipid homeostasis (APOA4) [43], regulation of extracellular matrix (ITIH4) [44] inflammation, and immune response (SERPINA 6, ITIH4). Furthermore, they are all affected in ocular disease [33] and also in AH [45]. The same analysis also revealed that treated RB eyes, with or without active tumors (Group 1, 2 and 4), showed an upregulation of proteins involved in metabolism, among those identified were mainly oxidoreductases (glutamate–cysteine ligase, transthyretin), transferases (glutathione transferase; glutaredoxin-1), and proteins involved in lipid metabolism (ApolipoproteinA4; ApolipoproteinA1). We found that in Group 4 (inactive RB), several of the upregulated proteins were involved in energy pathways. Some of these proteins mediate sensitivity towards chemotherapy [46]. Because cancer cells die and release their contents into the surrounding microenvironment following anticancer treatments, we speculate that a residual accumulation of these proteins persisted for a long time, even in eyes in remission after chemotherapy [47].

Our finding that AH from naïve RB presented a significant increase in proteins implicated in the regulation of nucleosides synthesis, compared to the other RB groups, could be explained by the fact that, in order to grow, neoplastic cells are highly dependent on the de novo synthesis of nucleotides to support DNA replication and the production of RNA [48].

Our finding of upregulated HIST2H2BE in the group with naïve RB was intriguing. Histones are nuclear proteins responsible for the structure of the nucleosome. They engage and intervene in chromatin structure and function through post-translational modifications [49,50]. Several mutations in H2B genes, especially in HIST1H2BC, HIST1H2BD, and HIST1H2BE, have been detected in cancer cells [51,52]. In humans, H2B are encoded by 11 genes and present 13 isoforms [53]. Among these isoforms, HIST2H2BE has been correlated with cancer aggressiveness [54] and implicated in tumor angiogenesis, cell proliferation, and invasiveness [55].

The presence of high levels of INADL/PATJ in the active aqueous (Group 2) and vitreous seeding (Group 1) groups may support the role of this protein in RB progression. INADL/PATJ is localized in the Golgi and/or nuclei and at the apical junction of the retinal pigment epithelium [56]. INADL is a scaffolding protein required for the formation of the tight junction that controls the correct development of apico-basal polarity in epithelial cells [57]. The altered function of apico-basal polarity leads to the disruption of cell–cell adhesions, cytoskeleton rearrangements, and loss of epithelial homeostasis [58]. This polarity alteration is a hallmark of cancer progression, since cancer cells may adopt this phenotype to become invasive and determine a metastatic disease [59].

Our finding of MIF to be highly expressed in active RB agrees with the results of a recent study showing that this protein, among others, is likely involved in the production of an immunosuppressive microenvironment in these intraocular tumors [23].

In Group 4 (inactive RB), we found proteins (such as Ube2V1) involved in protein catabolism. It has recently been observed that Ube2V1 supports epithelial mesenchymal transition (EMT) and metastasis, utilizing an autophagy-related mechanism [60]. Overall, this study provides the first proof of the principle that the composition of AH proteins may correlate with RB disease stage and holds the potential to identify clinically relevant biomarkers for RB management. As a preliminary approach, this study also shows some limitations. In particular, due to the small amount of biological material in the samples, all of the material was necessary for our proteomic analysis. Thus, it was not possible to validate the identified biomarkers in all samples using different approaches. Despite this fact, we validated the presence of selected proteins in cell lines derived from human RB tumors or vitreous seeding. Further investigations in a larger cohort of RB patients will determine the clinical significance of our initial findings (Supplementary Figure S1, Supplementary Materials, and Methods).

## 4. Materials and Methods

### 4.1. Subjects and Collection of AH

All included AH samples were collected from children with intraocular RB (*n* = 39) or with congenital cataracts (*n* = 5) treated in Jules Gonin Eye Hospital between September 2014 and October 2018. A total of 53 AH samples from 45 eyes of 44 different patients were analyzed. The samples could be categorized into 5 subgroups, depending on the type of disease and the clinical features of the tumor (if any) at the time of the anterior chamber paracentesis. Group 1 included samples from treated RB eyes with isolated vitreous relapse obtained at the time of the first intravitreal injection. Group 2 included samples from RB eyes with active aqueous seeding confirmed by cytopathology, with or without active tumors in other eye compartments. Group 3 included samples with naïve RB taken in vivo at the time of disease diagnosis or *ex vivo* after primary enucleation. Group 4 included samples from RB eyes in complete remission. Group 5 was the control group, with AH samples from eyes of healthy children with congenital cataracts.

For all patients, AH (0.1–0.15 mL) was obtained through an anterior chamber paracentesis performed with a 34 gauge needle [61] and immediately placed into a sterile collection tube, which was stored at −80 °C until the proteomic analysis was performed. For the RB patients with active disease treated conservatively, AH was collected just before an intraocular injection, as standard part of a previously described safety-enhanced technique for intravitreal [62] or intracameral chemotherapy, with the latter undergoing a triple freeze-and thaw cryo-coagulation of the entry point of the needle [61]. Samples from enucleated eyes were obtained just after the cut of the optic nerve. Samples from eyes with congenital cataracts were obtained per-operatively.

**Figure 4:**
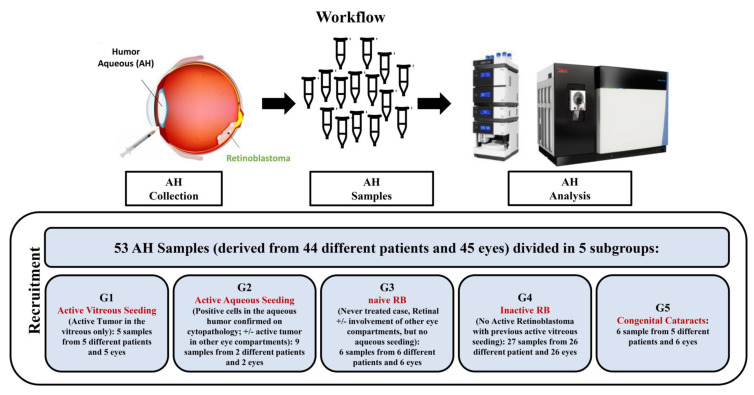
Workflow of the proteomic analysis of the aqueous humor in patients affected by retinoblastoma. The figure was created with https://biorender.com.

The number of samples in each subgroup and the clinical features at the time of the AH collection are summarized in Figure 4 and Supplementary Table S3, respectively.

### 4.2. Proteomic Setup

#### 4.2.1. Sample Preparation

The samples were processed using the in-StageTip [63] method, slightly modified [64]. Briefly, the pellets were re-suspended in 50 μL of lysis buffer (6M GdmCl, 10 mM TCEP, 40 mM CAA, 100 mM Tris pH 8.5). In Eppendorf, samples were denatured, reduced, alkylated, and lastly, 5% ProteaseMAX surfactant (Promega, Madison, WI, USA) was added to enhance protein digestion by providing a denaturing environment before the protease addition. The samples were added with 0.3 µg LysC and 0.7 µg trypsin in 250 µL of a dilution buffer (10% ACN, 25 mM Tris HCl pH 8.5) to dilute the ProteaseMAX to 0.1%. After overnight digestion at 37 °C, the peptides were acidified with 0.1% TFA and loaded into StageTip.

A tryptophan-based assay was performed on all samples, which was subsequently used as an inclusion criterion for the analysis. All samples below the threshold of 3 μg were used for the development of the method, running them as individual samples or as a pool, always specific for each group. The files have been integrated in the processing to maximize the identifications, but were not evaluated quantitatively and therefore, were neglected during the statistical processing.

#### 4.2.2. NanoLC Setup

The sample was loaded from the sample loop directly into the separation column, and the peptides were eluted with increasing organic solvent at a flow rate of 250 nL/min. The peptide separations were carried out at 60 °C using a 75 μm ID × 50 cm 2 μm, 100 Å C18 column-mounted in the thermostatic column compartment with a non-linear gradient of 5–45% solution B (80% CAN and 20% H_2_O, 5% DMSO, 0.1% FA) for 78 min.

#### 4.2.3. Mass Spectrometer Setup

The tryptic peptides were analyzed using an Orbitrap Fusion Tribrid mass spectrometer (Thermo Scientific Instruments, Bremen, Germany). Orbitrap detection was used for MS1 measurements at resolving powers of 120 K (at *m*/*z* 200). Data-dependent MS/MS analysis was performed in top speed mode with a 2 s. cycle-time, during which precursors detected within the range of *m*/*z* 375−1500 were selected for activation in order of charge state, using CHarge Ordered Parallel Ion aNalysis (CHOPIN). Briefly, if the precursor charge state was 2, then it was followed by CID fragmentation and by scanning in the ion trap, with an isolation window of 1.6 *m*/*z*, an AGC of 3e4, a maximum injection time of 250 ms, and a normalized collision energy of 35%. If the precursor charge state was 3–7 and the precursor intensity was greater than 500,000, then it was followed by HCD fragmentation and by scanning in the Orbitrap with a resolution of 15,000, an AGC of 1e4, and a maximum injection time 40 ms. If the precursor charge state was 3–7 and the precursor intensity was below 500,000, then it was followed by CID fragmentation and scanning in the ion trap.

#### 4.2.4. Data Analysis

Raw files were analyzed using MaxQuant software (version 1.6.10 and 1.6.14) and the MS/MS spectra were searched against the human FASTA file from the UniProt database (version 08/17), using a reverse decoy database and common contaminants (a list of 245 entries). The peptide search included cysteine carbamidomethylation as a fixed modification, and N-terminal acetylation and methionine oxidation as variable modifications. Trypsin was selected as the specified protease, and a maximum of two missed cleavages was allowed. The minimal peptide length was set to six amino acids, and the “match between runs” feature was enabled. A false discovery rate cutoff of 1% was applied at both the protein and PSM identification levels. The mass spectrometry data have been deposited to the ProteomeXchange Consortium via the PRIDE [65] partner repository with the dataset identifier PXD022218 and PXD022256. Reviewer account details: username: reviewer_pxd022218@ebi.ac.uk; password: afz0IYDk, and username: reviewer_pxd022256@ebi.ac.uk; password: YNhJVMks, respectively.

#### 4.2.5. Bioinformatic Analysis

All bioinformatics analyses were performed with the Perseus software [66]. Protein groups were filtered to require 70% valid values in at least one experimental group. The label-free intensities were expressed as base log2, and empty values were imputed with random numbers from a normal distribution for each column to best simulate low abundance values close to the noise level. A group was created for each clinical condition, and a t-tests were performed, with an s0 of 0.1 and a required a *p*-value of 0.05. To visualize the profile of the experiment, hierarchical clustering of the resulting proteins was performed with log2 intensities after z-score normalization of the data for each cell line, using Euclidean distances. The Venn diagram of identified proteins was calculated using the online tool jVenn [67].

#### 4.2.6. Open Target Analysis

This analysis generated a network containing 96 proteins associated with RB (source: Open Target) [24]. Through the graphic use of Cytoscape and of StringApp for the protein-protein interaction information, we have constructed the associated network shown in Figure 3. The nodes have dimensions related to the degree of connection, and the internal circumference shows the most connected proteins with a degree > 15. The outer circumference shows the less connected proteins (degree ≤ 15), and therefore, these are also of smaller dimensions. Connections are present only if the confidence level of the string is >0.4. The coloration of the nodes is a function of the average intensity of each group measured in mass spectrometry, normalized for graphic reasons on a scale of 1–100. The coloring and the percentage distribution of the node was done using the layout tools: Image/Chart of Cytoscape.

## 5. Conclusions

In conclusion, proteomic analysis of the AH of 47 RB samples and 6 controls allowed for the identification of a significant number of proteins in RB at various stages of the disease. This work provides a novel insight into the use of AH as a source of tumor biomarkers in this specific type of tumor. To our knowledge, this is the first proteomic study performed on AH from conservatively treated RB at different stages.

The characterization of the mechanisms underlying the clonal selection of the tumor cells and the subsequent seeding development still requires considerable study. However, this approach, among other liquid biopsy analyses, will undeniably contribute to unravel the disease tumorigenesis process and guide patient management in the future.

## Data Availability

The mass spectrometry data have been deposited to the ProteomeXchange Consortium via the PRIDE [Vizcaíno JA, 2016 update of the PRIDE database and related tools. Nucleic Acids Res] partner repository with the dataset identifier PXD022218 and PXD022256. Reviewer account details: username: reviewer_pxd022218@ebi.ac.uk; password: afz0IYDk, and username: reviewer_pxd022256@ebi.ac.uk; password: YNhJVMks, respectively. Restrictions apply to the availability of these data. The data will be public and accessible only with the code PXD022218 and PXD022256.

## Supplementary Materials

The following supporting information can be downloaded at: https://www.mdpi.com/article/10.3390/ijms232113458/s1.
