# Supplementary material for: Proteomics of Aqueous Humor as a Source of Disease Biomarkers in Retinoblastoma
Source: Int J Mol Sci. 2022 Nov 3;23(21):13458. doi: 10.3390/ijms232113458 (PMC9659039; doi:10.3390/ijms232113458)
Supplement: Supplementary file 1 [file ijms-23-13458-s001.zip › ijms-1931212-supplementary.pdf]

Supplementary Files:

Supplementary Table S1. Name and function of the top ten proteins differentially expressed between the different groups, by comparing two groups at the time.

Fold-change naïve RB (Group 3) vs congenital cataract (Group 5) n= 241	Protein ID	Gene name	Protein name	Protein Function
5,35673	A0A2R8YGX3	TPM4	Tropomyosin alpha-4 chain	Actin-binding, Muscle protein
4,85457	P04080	CSTB	Cystatin-B	Protease Binding
4,56122	P0C0L4	C4A	Complement C4-A	Endopeptidase inhibitor activity
4,22218	Q16778	HIST2H2BE	Histone H2B type 2-E	DNA Binding
4,18093	P62805	HIST1H4A	Histone H4	DNA Binding
4,16056	E9PB61	ALYREF	THO complex subunit 4	Chaperone
3,71647	H3BS70	ECI1	Enoyl-CoA delta isomerase 1	Isomerase; Acid fat metabolism
3,65666	J3QRJ3	THY1	Thy-1 antigen	Protein kinase binding
3,46058	Q9UBP9-3	GULP1	PTB domain-containing engulfment adapter protein 1	Phagocytosis, Engulfment
3,41917	Q12874	SF3A3	Splicing factor 3A subunit 3	RNA binding
-4,30656	Q9UII2	ATP5IF1	ATPase inhibitor	Enzyme inhibitor activity
-4,37824	Q13162	PRDX4	Peroxiredoxin-4	Thioredoxin peroxidase activity
-4,50416	Q9UHI8	ADAMTS1	A disintegrin and metalloproteinase with thrombospondin motifs 1	Metalloprotease
-4,64126	A0A2R8Y7M0	TMF1	TATA element modulatory factor	DNA-binding
-4,75289	A0A075B6K2	IGLV3-12	Immunoglobulin lambda variable 3-12	Antigen binding
-4,91635	Q7Z4T9-1	MAATS1	Cilia- and flagella-associated protein 91	Axonemal central apparatus assembly
-5,04124	Q9C0D2	CEP295	Centrosomal protein of 295 kDa	Microtubule binding
-5,59287	J3KSR8	SRSF1	Serine/arginine-rich-splicing factor 1	RNA-binding
-6,41151	P50991-2	CCT4	T-complex protein 1 subunit delta	Protein folding chaperone
-6,7774	A0A087WY08	THSD7A	Thrombospondin type-1 domain-containing protein 7A	Actin cytoskeleton reorganization
**Fold-change naïve RB (Group 3) vs inactive RB (Group 4) n=99**				
4,16987	Q16778	HIST2H2BE	Histone H2B type 2-E	DNA Binding
3,91358	P62805	HIST1H4A	Histone H4	DNA Binding
3,78363	K7EK07	H3F3B	Histone H3	DNA Binding
2,94542	P14174	MIF	Macrophage migration inhibitory factor	Protease binding
2,93107	A0A0A0MS09	IGHD	Immunoglobulin heavy constant delta	Antigen binding

2,60369	J3QRJ3	THY1	Thy-1 antigen	Protein kinase binding
2,58545	G3V3A0	SERPINA3	Alpha-1-antichymotrypsin	Serine-type endopeptidase inhibitor activity
2,47378	P01714	IGLV3-19	Immunoglobulin lambda variable 3-19	Antigen binding
2,47226	G3V3U4	PSMA6	Proteasome subunit alpha type	Endopeptidase activity
2,39743	P04080	CSTB	Cystatin-B	Protease Binding
-2,3841	P24592	IGFBP6	IGF-binding protein 6	Insulin-like growth factor I binding
-2,65906	Q15573	TAF1A	TBP-associated factor 1A	DNA binding
-2,72591	P06331	IGHV4-34	Immunoglobulin heavy variable 4-34	Antigen binding
-2,85974	Q5VU43-3	PDE4DIP	Myomegalin	Enzyme binding
-2,86199	Q9NPR2-2	SEMA4B	Semaphorin-4B	Semaphorin receptor binding
-3,42494	A0A2R8Y7M0	TMF1	TATA element modulatory factor	DNA-binding
-3,80541	P31944	CASP14	Caspase-14	cysteine-type endopeptidase activity involved in execution phase of apoptosis
-4,06714	A0A087WY08	THSD7A	THSD7A protein	Actin cytoskeleton reorganization
-4,54644	A0A0A0MSL3	UBE2V1	Ubiquitin-conjugating enzyme E2 variant 1	Regulation ubiquitination
-5,5349	O60826	CCDC22	Coiled-coil domain-containing protein 22	protein binding
**Fold-change inactive RB (Group 4) vs congenital cataract (Group 5) n=219**				
4,55489	Q9UBP9	GULP1	PTB domain-containing engulfment adapter protein 1	Phagocytosis, Engulfment
3,68028	P16520	GNB3	Transducin beta chain 3	GTPase binding
3,4783	Q86V81	ALYREF	THO complex subunit 4	chaperone
3,12474	P0C0L4	C4A	Complement C4-A	endopeptidase inhibitor activity
2,89213	Q14624	ITIH4	ITI heavy chain H4	endopeptidase inhibitor activity
2,87557	Q9BSC4	NOL10	Nucleolar protein 10	RNA binding
2,84919	P00738	HP	Haptoglobin	antioxidant activity
2,76883	Q9NQZ6	ZC4H2	Zinc finger C4H2 domain-containing protein	metal ion binding
2,66772	P08185	SERPINA6	Corticosteroid-binding globulin	steroid binding
2,62823	P0DJI8	SAA1	Serum amyloid A-1 protein	heparin binding
-2,81749	Q9UHI8	ADAMTS1	ADAM-TS 1	Metalloprotease
-2,99939	P84243	H3F3B	Histone H3.3	DNA binding
-3,11157	P18206	VCL	Vinculin	structural molecule activity
-3,22835	Q9C0D2	CEP295	Centrosomal protein of 295 kDa	microtubule binding
-3,29948	P30304	CDC25A	M-phase inducer phosphatase 1	protein kinase binding
-3,55858	Q07955	SRSF1	Serine/arginine-rich splicing factor 1	RNA-binding

-4,0664	Q8N3U4	STAG2	Cohesin subunit SA-2	chromatin binding
-4,31992	A0A075B6K2	IGLV3-12	Immunoglobulin lambda variable 3-12	antigen binding
-5,1572	P50991	CCT4	T-complex protein 1 subunit delta	protein folding chaperone
-6,13756	Q7Z4T9	MAATS1	Cilia- and flagella-associated protein 91	axonemal central apparatus assembly
**Fold-change active vitreous seeding (Group1) vs inactive RB (Group 4) n= 86**				
4,69686	Q8NI35-5	INADL/ PATJ	InaD-like protein	tight junction assembly
3,41233	E5RGQ7	DMTN	Dematin	actin binding
3,27358	Q16778	HIST2H2BE	Histone H2B type 2-E	DNA Binding
3,20713	E7EMB1	SWAP70	Switch-associated protein 70	DNA binding
2,84011	P49454	CENPF	Centromere protein F	microtubule binding
2,74007	E9PGC8	MAP1A	Microtubule-associated protein 1A	microtubule binding
2,32901	E7END6	PROC	Vitamin K-dependent protein C	calcium ion binding
2,30372	X6R3R3	SOGA1	Protein SOGA1	negative regulation of gluconeogenesis
2,20224	Q8NEG4	FAM83F	Protein FAM83F	protein kinase binding
2,18047	A0A5H1ZRQ7	IGLC7	Immunoglobulin lambda constant 7	antigen binding
-2,55872	Q8WYP5	AHCTF1	Protein ELYS	DNA binding
-2,66037	Q8TE73	DNAH5	Dynein axonemal heavy chain 5	ATP binding
-2,68194	P45973	CBX5	Chromobox protein homolog 5	Protein binding
-2,84219	Q9NQZ6-2	ZC4H2	Zinc finger C4H2 domain-containing protein	metal ion binding
-2,85539	P10451-5	SPP1	Osteopontin	extracellular matrix binding
-3,04056	A0A087WVF7	ITSN2	Intersectin-2	calcium ion binding
-3,23823	Q5HYK7-3	SH3D19	SH3 domain-containing protein 19	cytoskeleton organization
-3,25443	P20851	C4BPB	C4b-binding protein beta chain	regulation of opsonization
-3,63481	Q9C0D2	CEP295	Centrosomal protein of 295 kDa	microtubule binding
-4,74572	A0A0A0MSL3	UBE2V1	Ubiquitin-conjugating enzyme E2 variant 1	Regulation ubiquitination
**Fold-change active aqueous seeding (Group 2) Vs naïve RB (Group 3) n=131**				
7,33912	P02787	TF	Serotransferrin	transferrin receptor binding
7,00994	Q8NI35-5	INADL	InaD-like protein	tight junction assembly
5,65275	P31944	CASP14	Caspase-14	cysteine-type endopeptidase activity involved in execution phase of apoptosis
4,61768	Q9HCQ7-2	NPVF	Neuropeptide RFRP-3	signaling receptor binding
3,94207	H7C5C6	STIM2	Stromal interaction molecule 2	calcium ion binding
3,67324	Q8NFU5	IPMK	Inositol polyphosphate multikinase	transferase
3,63418	Q9Y383	LUC7L2	Putative RNA-binding protein Luc7-like 2	RNA binding
3,48703	Q5VU43-3	PDE4DIP	Myomegalin	enzyme binding

3,44613	Q8IWA6	CCDC60	Coiled-coil domain-containing protein 60	Microtubule-binding protein
3,40074	F2Z2G5	WFDC3	WAP four-disulfide core domain protein 3	serine-type endopeptidase inhibitor activity
-3,06905	Q5HYK7-3	SH3D19	SH3 domain-containing protein 19	cytoskeleton organization
-3,11417	A0A075B6H9	IGLV4-69	Immunoglobulin lambda variable 4-69	antigen binding
-3,3074	G3V3A0	SERPINA3	Alpha-1-antichymotrypsin	serine-type endopeptidase inhibitor activity
-3,40435	P02766	TTR	Transthyretin	protein-containing complex binding
-3,46545	A0A075B6I9	IGLV7-46	Immunoglobulin lambda variable 7-46	antigen binding
-3,57237	A0A0A0MSS5	SMARCA2	Probable global transcription activator SNF2L2	DNA-binding
-3,82615	P48507-2	GCLM	Glutamate--cysteine ligase regulatory subunit	protein-containing complex bindin
-4,039	A0A0C4DGD1	CAST	Calpain inhibitor	endopeptidase inhibitor activity
-4,70627	Q5KU26	COLEC12	Collectin-12	metal ion binding
-4,85584	E9PB61	ALYREF	THO complex subunit 4	chaperone

Supplementary Table S2. Lyst of 96 genes coding proteins represented in the network with overall association score.

Symbol	Overall Association Score	Name
TUBB3	0,413198	Tubulin beta 3 class III
TUBB4B	0,406135	Tubulin beta 4B class IVb
TUBB	0,406135	Tubulin beta class I
AKT1	0,086406	AKT serine/threonine kinase 1
PPIA	0,073916	Peptidylprolyl isomerase A
LRG1	0,057285	Leucine rich alpha-2-glycoprotein 1
MMP2	0,040225	Matrix metallopeptidase 2
PRDX1	0,035627	Peroxiredoxin 1
TP53BP2	0,033624	Tumor protein p53 binding protein 2
ESD	0,032303	Esterase D
SERPINF1	0,032091	Serpin family F member 1
WIF1	0,031703	WNT inhibitory factor 1
S100A4	0,030771	S100 calcium binding protein A4
ENO2	0,029371	Enolase 2
ENO1	0,028694	Enolase 1
APEX1	0,028458	Apyrimidinic endodeoxyribonuclease 1
NME1	0,027349	NME/NM23 nucleoside diphosphate kinase 1
RBP3	0,025557	Retinol binding protein 3
ILF3	0,025131	Interleukin enhancer binding factor 3
CDH2	0,020569	Cadherin 2
TGFBI	0,01774	Transforming growth factor beta induced
TKT	0,017029	Transketolase
ECM1	0,017001	Extracellular matrix protein 1

STMN1	0,0165	Stathmin 1
SMARCA2	0,016303	SWI/SNF relatedmember 2
GAPDH	0,015769	Glyceraldehyde-3-phosphate dehydrogenase
HNRNPK	0,015317	Heterogeneous nuclear ribonucleoprotein K
HMGB1	0,014793	High mobility group box 1
PTPRC	0,014414	Protein tyrosine phosphatase receptor type C
NCAM1	0,014044	Neural cell adhesion molecule 1
MCM7	0,014044	Minichromosome maintenance complex component 7
ACTB	0,013654	Actin beta
APOD	0,013305	Apolipoprotein D
RELN	0,012935	Reelin
CLU	0,012935	Clusterin
LAMB2	0,012165	Laminin subunit beta 2
CDC25A	0,01121	Cell division cycle 25°
TUBA1B	0,011087	Tubulin alpha 1b
RCVRN	0,010595	Recoverin
CD44	0,010502	CD44 molecule
SFRP2	0,009424	Secreted frizzled related protein 2
VIM	0,009138	Vimentin
TMPO	0,00887	Thymopoietin
EGFLAM	0,00887	EGF like, fibronectin type III and laminin G domains
IGFBP5	0,00887	Insulin like growth factor binding protein 5
AHSG	0,008131	Alpha 2-HS glycoprotein
CRYAB	0,007946	Crystallin alpha B
LDHA	0,007925	Lactate dehydrogenase A
IGFBP2	0,007761	Insulin like growth factor binding protein 2
COL18A1	0,007392	Collagen type XVIII alpha 1 chain
NR1I2	0,007392	Nuclear receptor subfamily 1 group I member 2
FEN1	0,007392	Flap structure-specific endonuclease 1
TGFB2	0,007392	Transforming growth factor beta 2
CLUL1	0,007392	Clusterin like 1
MYH9	0,007022	Myosin heavy chain 9
SOD1	0,005913	Superoxide dismutase 1
ANXA5	0,005391	Annexin A5
IGFBP3	0,004805	Insulin like growth factor binding protein 3
LOX	0,004435	Lysyl oxidase
FN1	0,004322	Fibronectin 1
HSPA4	0,00425	Heat shock protein family A (Hsp70) member 4
HMGB2	0,004065	High mobility group box 2
LMNB1	0,004065	Lamin B1
UCHL1	0,003696	Ubiquitin C-terminal hydrolase L1
CENPE	0,003696	Centromere protein E
SLC2A1	0,003696	Solute carrier family 2 member 1
RAD50	0,003696	RAD50 double strand break repair protein
CHGA	0,003696	Chromogranin A
NCL	0,003696	Nucleolin
RS1	0,003696	Retinoschisin 1
MCM6	0,002957	Minichromosome maintenance complex component 6
C3	0,002217	Complement C3
MYOC	0,002217	Myocilin

CANX	0,002217	Calnexin
SPP1	0,002217	Secreted phosphoprotein 1
PPA1	0,002217	Inorganic pyrophosphatase 1
APOE	0,002217	Apolipoprotein E
APOA1	0,002217	Apolipoprotein A1
TIMP1	0,002217	TIMP metallopeptidase inhibitor 1
PON1	0,002217	Paraoxonase 1
AGT	0,002217	Angiotensinogen
PRTN3	0,001478	Proteinase 3
KMT2C	0,001478	Lysine methyltransferase 2C
CFH	0,001478	Complement factor H
RBBP4	0,001478	RB binding protein 4, chromatin remodeling factor
SERPINA3	0,001478	Serpin family A member 3
GBE1	0,001478	1,4-alpha-glucan branching enzyme 1
DCD	0,001478	Dermcidin
HTRA1	0,001478	HtrA serine peptidase 1
APP	0,001478	Amyloid beta precursor protein
MCM3	0,001478	Minichromosome maintenance complex component 3
MAPK1	0,001478	Mitogen-activated protein kinase 1
KHSRP	0,001478	KH-type splicing regulatory protein
CYCS	0,001478	Cytochrome c, somatic
ALYREF	0,001478	Aly/REF export factor
VGF	0,001478	VGF nerve growth factor inducible

Supplementary Figure S1:

The expression of the 5 most significantly upregulated proteins in the AH samples with RB when compared to the control group (namely PATJ in Group 1 and 2, Histone H2B type 2-E in Group 3 and Ube2V1 in Group 4, GAPDH and CD44 in all RB disease group) was analyzed for clinical validation and confirmed by a Western blot analysis performed in RB primary cell lines derived from retinal and vitreous tumor (Figure 4).

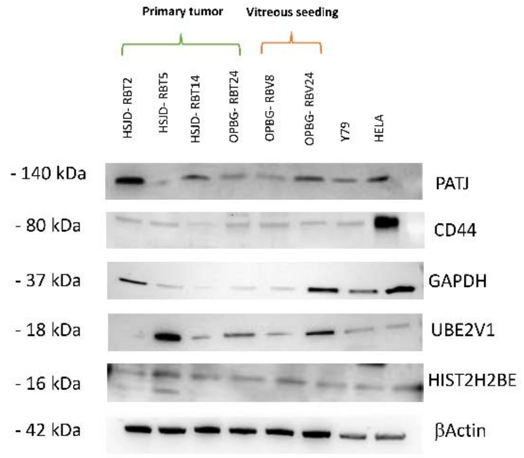

Western blot analysis of 5 selected proteins: PatJ; CD44; GAPDH; UNE2V1 and HIST2H2BE in 4 RBT and 2 RBVS representative RB cell lines. β-Actin was reported as loading control.

Supplementary Table S3. Patients' demographics, previous given treatments and clinical features at time of the aqueous humor (AH) sampling.

Patient/ Study Group	Serie Samples	Age at AH sampling (months)	Gender	Laterality	Studied Eye	Classification Group (IIRC)	Previous treatments	Tumor status at time of AH collection	Study Group
**1 /1**	S001	36	F	U	OS	D	IVC (2x), Thermo-CT (1x) IAC (3x) FT	Active VS *	**GROUP 1 Isolated active vitreous seeding**
**2/1**	S002	71	M	U	OD	D	IVC (6x), IAC M (6x), IAC MT (3x), FT	Active VS *	
**3/1**	S003	30	M	B	OD	D	IVC (2x), IAC M (1x), IAC MT (2x), Ivit M (3x), FT	Active VS *	
**4/1**	S004	33	M	B	OD	D	IVT (2x), IAC M (3x), IAC MT (2x), Ivit T (1x), FT	Active VS *	
**5/1**	S005	77	F	U	OD	D	IAC M (2x), FT	Active VS *	
**6/2**	S006	255	M	B	OS	na	IVC (13x), FT, plaque (1x), IAC M(6x), Ivit M (x, elsewhere)	Active AS §	**GROUP 2 Active aqueous humor seeding ± other intraocular active sites**
**6/2**	S007	255	M	B	OS	na	IVC (13x), FT, plaque (1x), IAC M(6x), Ivit M (x elsewhere), ICC (1x), IvitM (1x)	Active AS §	
**6/2**	S008	256	M	B	OS	na	IVC (13x), FT, plaque (1x), IAC M(6x), Ivit M (x elsewhere), ICC (4x), IvitM (3x)	Active AS §	
**6/2**	S009	256	M	B	OS	na	IVC (13x), FT,	Active AS §	

							plaque (1x), IAC M(6x), Ivit M (x elsewhere), ICC(5x), Ivit M(4x)		
**6/2**	S010	256	M	B	OS	na	IVC (13x), FT, plaque (1x), IAC M(6x), Ivit M (x elsewhere), ICC (6x), Ivit M(5x)	Active AS §	
**6/2**	S011	256	M	B	OS	na	IVC (13x), FT, plaque (1x), IAC M(6x), Ivit M (x elsewhere), ICC (7x), Ivit M(5x)	Active AS §	
**6/2**	S012	257	M	B	OS	na	IVC (13x), FT, plaque (1x), IAC M(6x), Ivit M (x elsewhere), ICC M (9x) Ivit M (7x)	Active AS §	
**7/2**	S013	113	M	U	OS	D	-	Active AS §	
**7/2**	S014	113	M	U	OS	D	Ivit T (1x), ICC T (1x), IAC M(1x)	Active AS §	
**8/3**	S015	3	F	U	OS	E	-	Active RT + SRS	**GROUP 3 naïve RB**
**9/3**	S016	28	F	U	OS	E	-	Active RT + VS °	
**10/3**	S017	20	F	U	OD	E	-	Active RT °	
**11/3**	S018	47	M	U	OS	E	-	Active RT + VS °	
**12/3**	S019	624	F	U	OS	E	-	Active RT + VS °	
**13/3**	S020	61	M	U	OD	E	-	Active RT + VS °	
**14/4**	S021	16	M	B	OD	na	IVC (4x), FT, IAC M (3x), IvitC M (3x)	Inactive §	**GROUP 4: inactive RB**
**15/4**	S022	17	M	U	OD	E	IVC (3x), FT, Ivit M (2x)	Inactive §	
**16/4**	S023	10	F	B	OD	C	IVC (3x), FT, IvitC M (2x)	Inactive §	
**17/4**	S024	36	F	U	OS	D	IAC M (3x), IvitC M (5x)	Inactive §	

**18/4**	S025	61	M	U	OS	D	IVC (2x),<br>ICC (6x),<br>IvitC M (7x)	Inactive §
**19/4**	S026	101	F	B	OD	D	IVC (2x),<br>EBR (2x),<br>IvitC M (9x),<br>Plaque (1x),<br>FT	Inactive §
**20/4**	S027	56	M	U	OS	na	IAC MT (8x),<br>Thermo-CT<br>(2x),<br>Ivit (6x),<br>FT,<br>Plaque (1x),<br>Ivit anti-VEGF<br>(1x)	Inactive §
**21/4**	S028	42	F	U	OS	D	IVC (1x),<br>IAC M (3x),<br>FT,<br>IvitC M (4x)	Inactive §
**21/4**	S029	49	F	U	OS	D	IVC (1x),<br>IAC M (3x),<br>FT,<br>Plaque (1x),<br>IvitC M (6x)	Inactive §
**22/4**	S030	50	M	U	OS	D	IVC (na),<br>IAC M (3x),<br>FT,<br>IvitC M (1x)	Inactive §
**23/4**	S031	36	F	U	OS	D	IVC (2x),<br>Thermo-CT (1),<br>FT,<br>IAC M (3x),<br>IvitC M (3x)	Inactive §
**24/4**	S032	38	M	U	OS	D	IAC M (3x),<br>IvitC M (6x)	Inactive §
**25/4**	S033	49	M	B	OD	D	IVC (2x),<br>Thermo CT<br>(2x),<br>FT,<br>IvitC M (10x),	Inactive §§
**26/4**	S034	95	M	U	OS	na	IAC M (3x),<br>IvitC M(6x)	Inactive §§
**27/4**	S035	75	F	U	OD	na	IVC (4x),<br>EBR (1x),<br>IvitC M (6x)	Inactive §§
**28/4**	S036	16	M	B	OS	B	IVC (6x),<br>FT,<br>IAC M (1x),<br>IvitC M (1x)	Inactive §
**29/4**	S037	35	M	U	OS	D	IVC (2x),<br>IAC M (3x),	Inactive §

							FT, IvitC M (1x)		
**30/4**	S038	61	F	U	OD	E	IAC M (3x), FT, Ivit M (6x)	Inactive [§]	
**31/4**	S039	7	F	B	OS	B	IVC (2x), FT, IvitC M (2x)	Inactive [§]	
**32/4**	S040	31	F	U	OD	D	IVC (4x), IvitC M (5x), FT, IAC M (2x)	Inactive [§]	
**33/4**	S041	24	M	U	OS	D	IVC (1x), IAC M (3x), Ivit M (3x)	Inactive [§]	
**34/4**	S042	37	F	B	OS	D	IAC M (3x), FT, IvitC M (3x)	Inactive [§]	
**35/4**	S043	38	M	U	OS	C	IVC (1x), IAC M (1x), IAC M+T (1x), IvitC M (4x), FT, plaque (1x)	Inactive [§]	
**36/4**	S044	41	F	U	OS	D	IVC (1x), IAC M (2x), IvitC M (1x)	Inactive [§]	
**37/4**	S045	12	M	B	OD	D	IVC (2x), FT, IvitC M (2x)	Inactive [§]	
**38/4**	S046	30	M	B	OS	D	IVC (2x), Thermo CT (1x), FT, IAC M (3x), IVC M+T (2x), IvitC M (4x), plaque (1x)	Inactive [§]	
**39/4**	S047	20	M	U	OS	D	IAC M (3x), IvitC M (2x)	Inactive [§]	
**40/5**	S048	na	M	B	OS		-		**GROUP 5 congenital cataract**
**41/5**	S049	na	M	B	OS		-		
**42/5**	S050	65	M	B	OD		-		
**43/5**	S051	na	F	B	OS		-		
**42/5**	S052	65	M	B	OS		-		
**44/5**	S053	99	M	U	OS		-		

Legends: M = male; F = female; U = unilateral; B = bilateral; IIRC = International Intraocular Retinoblastoma Classification (Linn Murphree, 2005); OS = oculus sinister; OD = oculus dexter; na = not available; IVC = intravenous chemotherapy; Thermo-CT = Thermochemotherapy; FT: focal laser treatment; IAC = intraarterial chemotherapy; IvitC =intravitreal chemotherapy; ICC = intracameral chemotherapy; M = melphalan; T = topotecan; EBR = external beam radiotherapy; VS = vitreous seeding; AS = aqueous seeding; SRS = subretinal seeding; *= AH sample obtained at time of the first IvitC given for an isolated vitreous disease relapse: § = confirmed on cytopathology;°= AH sample obtained after primary enucleation; § = AH sampling obtained at time of a consolidation IvitC injection, with no other intraocular active site;§§ = obtained at time of cataract surgery with no intraocular active site

Supplementary Materials and methods:

Cells and Cell Culture:

Human RB cell lines HSJD-RBT-2, HSJD-RBT-5, HSJD-RBT-14 were established from enucleated eyes of RB patients at Hospital Sant Joan de Deu (HSJD, Barcelona, Spain). Specimens were collected from two possible sources: solid tumor tissue in the retina (RB tumor; RBT) [Pascual-Pasto, G. *et al.* Preclinical platform of retinoblastoma xenografts recapitulating human disease and molecular markers of dissemination. *Cancer Lett.* **2016**, *1*, 10-19. doi: 10.1016/j.canlet.2016.06.012]. Human RB cell lines OPBG-RBVS8 and OPBG-RBT24 and OPBG-RBVS 24 were obtained from enucleated eyes of RB Patients at Bambino Gesù Children Hospital. Cells were cultured as floating tumor-spheres in neural stem cell medium (serum-free), consisting of Neurobasal-A Medium (1×, ThermoFisher, Waltham, MA, USA), plus DMEM/F-12 (1×, ThermoFisher, Waltham, MA, USA) supplemented with HEPES Buffer Solution (1M, ThermoFisher, Waltham, MA, USA), Sodium pyruvate mem 100 mM (CE, ThermoFisher, Waltham, MA, USA), MEM Non-essential Amino Acids Solution 10 mM (100×, ThermoFisher, Waltham, MA, USA), GlutaMAX-I Supplement (100×, ThermoFisher, Waltham, MA, USA), Antibiotic-Antimycotic (100×, ThermoFisher, Waltham, MA, USA) B27 Supplement Minus Vitamin-A (50×, ThermoFisher, Waltham, MA, USA), Recombinant Human-basic FGF (20 ng/mL, Peprotech, Cranbury, NJ, USA), Recombinat Human EGF (20 ng/mL, Peprotech, Cranbury, NJ, USA), Human PDGF-AA (20ng/mL, Peprotech, Cranbury, NJ, USA), Human PDGF-BB (10 ng/mL, Peprotech, Cranbury, NJ, USA) and Heparin Solution 0.2% (2 µg/mL, Sigma Aldrich, St. Louis, MO, USA), as previously described [Monje, M. *et al.* Hedgehog-responsive candidate cell of origin for diffuse intrinsic pontine glioma. *Proc Natl Acad Sci U S A.* **2011,** *11*, 4453-4458. doi: 10.1073/pnas.1101657108]. Cell lines were maintained at 37 °C in a 5% (v/v) CO_2 humidified incubator.

Western Blot:

Cell lysis buffer (Cell Lysis Buffer (10×) #9803 Cell Signalling Technology, Danvers, MA, USA), supplemented with 10 mM phenylmethylsulphonylfluoride (PMSF 93,482 Sigma, St. Louis, MO, USA), as a protease inhibitor was used to extract the proteins from cells. Briefly, lysates were incubated on ice for 15 min and centrifugated at 13,000× g for 20 min at 4 °C. Equal micrograms of proteins, quantified with BCA assay (Pierce, Thermo Scientific, Waltham, MA, USA) and boiled in SDS-loading buffer (2× Laemmli Sample Buffer BIORAD cat.#161-0737), were resolved on 10% SDS-PAGE and transferred to PVDF membranes (Immun-Blot® PVDF Membrane for protein Blotting BIORAD cat.#162-0177). Blots were blocked for 1 h in TBS-T (TBS plus 0.05% Tween-20), 5% nonfat, dried milk and probed overnight at 4 °C with anti-GAPDH (D16H11) XP (Cell Signaling Technology®, Danvers, MA, USA), anti-UBE2V1(HPA052535) (ATLAS ANTIBODIES), Anti-PATJ (PA5-76397) (ThermoFisher Scientific, Waltham, MA, USA), Anti-Histone H2B type 2-E (H2be) (ABE1384) (Merck, Millipore), CD44 (156-33C11) (Cell Signalling Technology) orβ-Actin (C4) (sc-47778) (Santa Cruz Biotechnology, Dallas, TX, USA). Immunocomplexes were detected with horseradish peroxidase-conjugated species-specific secondary antibodies (Santa Cruz Biotechnology, Dallas, TX, USA), followed by enhanced chemiluminescence reaction with Immobilion Western Chemiluminescence HRP substrate WBKLS0100 (Millipore, Burlington, MA, USA).
